# Linking photosynthesis and yield reveals a strategy to improve light use efficiency in a climbing bean breeding population

**DOI:** 10.1093/jxb/erad416

**Published:** 2023-10-25

**Authors:** Beat Keller, Jonatan Soto, Angelina Steier, Ana Elisabeth Portilla-Benavides, Bodo Raatz, Bruno Studer, Achim Walter, Onno Muller, Milan O Urban

**Affiliations:** Crop Science, Institute of Agricultural Sciences, ETH Zurich, Zurich, Switzerland; Molecular Plant Breeding, Institute of Agricultural Sciences, ETH Zurich, Zurich, Switzerland; Bean Program, Crops for nutrition and health, International Center for Tropical Agriculture (CIAT), Cali, Colombia; Institute of Bio- and Geosciences, IBG-2: Plant Sciences, Forschungszentrum Jülich GmbH, Jülich, Germany; Bean Program, Crops for nutrition and health, International Center for Tropical Agriculture (CIAT), Cali, Colombia; Bean Program, Crops for nutrition and health, International Center for Tropical Agriculture (CIAT), Cali, Colombia; Molecular Plant Breeding, Institute of Agricultural Sciences, ETH Zurich, Zurich, Switzerland; Crop Science, Institute of Agricultural Sciences, ETH Zurich, Zurich, Switzerland; Institute of Bio- and Geosciences, IBG-2: Plant Sciences, Forschungszentrum Jülich GmbH, Jülich, Germany; Bean Program, Crops for nutrition and health, International Center for Tropical Agriculture (CIAT), Cali, Colombia; University of Cambridge, UK

**Keywords:** Breeding, bean, light use efficiency, photosynthesis, phenotyping, selection

## Abstract

Photosynthesis drives plant physiology, biomass accumulation, and yield. Photosynthetic efficiency, specifically the operating efficiency of PSII (*F*_q_'/*F*_m_'), is highly responsive to actual growth conditions, especially to fluctuating photosynthetic photon fluence rate (PPFR). Under field conditions, plants constantly balance energy uptake to optimize growth. The dynamic regulation complicates the quantification of cumulative photochemical energy uptake based on the intercepted solar energy, its transduction into biomass, and the identification of efficient breeding lines. Here, we show significant effects on biomass related to genetic variation in photosynthetic efficiency of 178 climbing bean (*Phaseolus vulgaris* L.) lines. Under fluctuating conditions, the *F*_q_'/*F*_m_' was monitored throughout the growing period using hand-held and automated chlorophyll fluorescence phenotyping. The seasonal response of *F*_q_'/*F*_m_' to PPFR (Response_G:PPFR_) achieved significant correlations with biomass and yield, ranging from 0.33 to 0.35 and from 0.22 to 0.31 in two glasshouse and three field trials, respectively. Phenomic yield prediction outperformed genomic predictions for new environments in four trials under different growing conditions. Investigating genetic control over photosynthesis, one single nucleotide polymorphism (Chr09_37766289_13052) on chromosome 9 was significantly associated with Response_G:PPFR_ in proximity to a candidate gene controlling chloroplast thylakoid formation. In conclusion, photosynthetic screening facilitates and accelerates selection for high yield potential.

## Teaser

Automated photosynthesis phenotyping outperformed genomic predictions, identifying high-yielding climbing bean lines and a candidate gene for high photosynthetic performance.

## Introduction

Photosynthesis is highly responsive to fluctuating environmental conditions, constantly adjusting solar energy uptake to optimize growth (Demmig-Adams *et al*., 2012). Therefore, photosynthetic performance reflects growth conditions and the plant’s ability to cope with these conditions (Murchie *et al.*, 2018). The highly dynamic response of photosynthesis to changing environmental conditions challenges the true estimation of photosynthetic performance in temporal and spatial dimensions (i.e. diurnally up to the growth period and within the individual plant canopy up to heterogeneities in the field, respectively) (Kaiser *et al.*, 2018). Nevertheless, photosynthetic parameters have successfully predicted biomass and yield in crops and breeding lines (Fischer *et al.*, 1998; Lopez *et al.*, 2019; Keller *et al.*, 2022b). Unfortunately, these parameters have rarely been exploited. Is photosynthetic performance a limiting factor for yield? Can photosynthetic traits be used as a breeding target to increase yield (Gifford and Evans, 1981; Sinclair *et al.*, 2019)?

### Photosynthesis for yield improvement

Photosynthetic efficiency continues to be a focus for crop yield improvement research because conventional traits, such as harvest index, are already highly exploited (Reynolds *et al.*, 2012; Long *et al.*, 2015). Although photosynthetic performance has often improved in high-yielding lines, remaining genetic variation suggests further potential for improvement (Driever *et al.*, 2014; Theeuwen *et al.*, 2022). When targeting photosynthetic traits for crop improvement, there are two main strategies: (i) optimizing the photosynthetic pathway through genetic engineering, or (ii) selecting breeding lines with higher photosynthetic efficiency under apparent growing conditions (Parry *et al.*, 2011). The former strategy, aimed at improving the photosynthetic processes, has been successful in field trials in tobacco, rice, and soybean (Kromdijk *et al.*, 2016; South *et al.*, 2019; López-Calcagno *et al.*, 2020; De Souza et al., 2022). The latter strategy, which aims to increase photosynthetic efficiency by selecting adapted lines to growing conditions, produced some striking results. For example, Fischer *et al.* (1998) reported a linear yield increase comparing eight wheat (*Triticum aestivum* L.) varieties released between 1962 and 1988. The yield increase (+27%) was associated with 23% and 63% increases in the maximum photosynthetic rate (*A*_max_) and stomatal conductance (*g*_s_), respectively. Another study reported a correlation of *r*=0.57 between steady-state spikelet photosynthesis and biomass using 12 contrasting wheat breeding lines (Molero and Reynolds, 2020). In soybean, photosynthesis and grain yield were strongly correlated (*r*=0.8) in 383 lines (Lopez *et al.*, 2019). Strong correlations between biomass and photosynthesis measured by gas exchange have also been shown in earlier studies in wheat and soybean (Ashley and Boerma, 1989; Gutiérrez-Rodríguez *et al.*, 2000; Carmo-Silva *et al.*, 2017). In many other studies reviewed by Zelitch (1982), a relationship between photosynthesis and yield was not observed because the photosynthetic performance was quantified over a short period that was not representative of the entire fluctuating growing season.

### Photosynthesis under fluctuating conditions

The energy transduction from incident solar radiation to biomass involves the interception of photosynthetically active radiation by the leaf canopy, conversion of radiant energy to photochemical energy, and transduction into biomass (Zhu *et al.*, 2010). The light energy intercepted by plant leaves is transmitted, reflected, and absorbed depending on the pigment composition (Porcar-Castell *et al*., 2014). The absorbed solar energy is then dynamically partitioned between three different pathways (Butler, 1978). Photosynthesis and dissipation of excess energy as heat through non-photochemical quenching (NPQ) are two pathways that are physiologically regulated to optimize plant growth and development (Butler, 1978; Bilger and Bjorkman, 1990). Approximately 2–4% of the absorbed energy is re-emitted as chlorophyll fluorescence (ChlF) via the third pathway (Porcar-Castell *et al*., 2014). Significant losses occur through NPQ (up to 50% over the growing season), preventing damage and determining whole-canopy photosynthesis (Ishida *et al.*, 2014; Murchie and Ruban, 2020). The changing light intensity during the day together with the NPQ response causes the typical diurnal pattern of photosynthesis (Pieruschka *et al*., 2010; Ishida *et al.*, 2014; Keller *et al.*, 2019a). This highly dynamic response of photosynthesis requires long-term monitoring to determine photosynthetic efficiency throughout the season. Photosynthesis measurements via CO_2_ assimilation over longer growing periods are time consuming—even when the photochemical uptake is approximated by variables such as *A*_max_, which are usually derived under high light intensities around midday. In addition, the photosynthetic capacity may vary in different leave types, as shown for wheat (Salter *et al.*, 2020). This may explain why such screenings have not been further implemented in breeding programs when phenotypic information from thousands of lines is required. In contrast to gas exchange measurements, the operating efficiency of PSII (*F*_q_'/*F*_m_') can be obtained in seconds and is widely used in plant physiology as a proxy for growth performance (Kalaji *et al.*, 2016; Murchie *et al.*, 2018). It is determined by the modulated ChlF signal, which describes the proportion of energy quantum used for electron transport (ET) from the absorbed photosynthetic photon fluence rate (PPFR) (Schreiber *et al.*, 1986; Baker, 2008). The ET at PSII is linearly related to CO_2_ assimilation in the absence of photorespiration or cyclic ET (Genty *et al.*, 1989; Yamori *et al.*, 2012). Photorespiration and cyclic ET are increased, for example, under heat and drought stress conditions (Golding *et al.*, 2004; Zhang and Sharkey, 2009; Hermida-Carrera *et al.*, 2016; Yamori and Shikanai, 2016). This method is suitable for rapid, non-invasive, long-term observation of linear ET, approximating the energy conversion efficiency of intercepted PPFR to photochemical energy (ϵ_e_), as well as the competing NPQ (Demmig-Adams *et al.*, 2012; Keller *et al.*, 2022b).

### Early genomic and phenomic selection

Genetic gain depends on high selection intensity on a heritable trait in short breeding cycles. The selection of a trait can be aided by genomic or phenomic estimates. Genomic predictions allow calculation of a genomic estimated breeding value (GEBV) from seed DNA samples, enabling selection before planting (Meuwissen *et al.*, 2001). However, many breeding programs require cheap and rapid selection methods at early stages of a selection process because genotyping of thousands of breeding lines is expensive (Furbank *et al.*, 2019). Phenomic selection uses high-throughput measurements, such as the spectral or ChlF signal, to predict a target trait. This also favors adaptation to new and future environments, accounting for genotype by environment interaction (G×E). The G×E can then be described by the interaction between genotype and the covariate (G×Ec), which is measured for each line in each environment (Eeuwijk *et al.*, 2016). Therefore, if measurements between lines are sufficiently correlated with the target trait, and are cheap and precise, phenomic selection is an efficient alternative to genomic selection.

Recently, ChlF measurements were shown to estimate biomass in 12 maize (*Zea mays* L.) and nine soybean [*Glycine max* (L.) Merr.] lines under field and naturally fluctuating conditions using automated high-throughput phenotyping (Keller *et al.*, 2022b). This approach could be modified to screen large numbers of early breeding lines for genetic variability in photosynthetic performance. This is especially useful in crops with longer growth cycles, such as the climber types of common bean (*Phaseolus vulgaris* L.). Photosynthetic performance is especially interesting in legumes, because they counteract protein dilution when assimilating more CO_2_ by increasing biological nitrogen fixation for protein synthesis (Evans and Clarke, 2019).

To study the relationship between photosynthetic traits and biomass production in climbing bean breeding lines, we investigated four hypotheses: (i) photosynthetic performance over the growing season is correlated with biomass production; (ii) selection based on the ϵ_e_ can increase the selection effectiveness of higher grain yield lines; (iii) photosynthesis-related traits (determined under the specific growing conditions) are more accurate than genomic prediction to predict yield of new lines in new environments; and (iv) genes contributing in the control of photosynthesis can be identified using genome-wide association study (GWAS).

## Materials and methods

### Plant material

This study used 178 breeding lines out of the complete climbing bean panel (VEC, total 290 lines) genotyped by Keller *et al.* (2022a). The panel represents the genetic variation of the climbing bean breeding program at the International Center for Tropical Agriculture (CIAT). Most of the lines belong to the Andean gene pool. A few lines are admixtures between the Andean and Mesoamerican gene pools.

### Growth conditions

#### Glasshouse

**Fig. 1.**
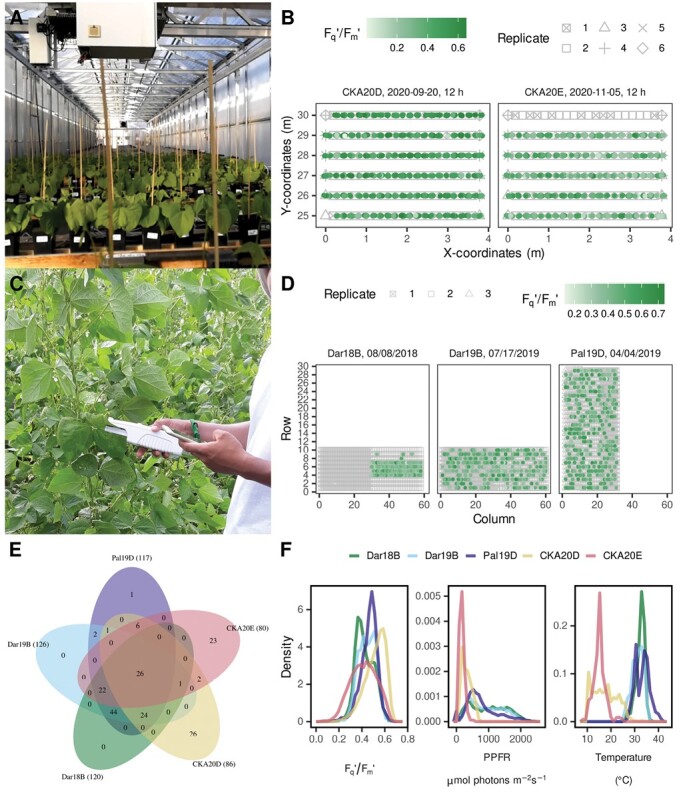
Photosynthesis measurements based on probed chlorophyll fluorescence (ChlF) of climbing bean lines in the glasshouse and field. (A) Scans in the glasshouse were carried out by an automated light-induced fluorescence transient (LIFT) system under fluctuating conditions. (B) Climbing bean lines were arranged in pots (gray symbols) and the operating efficiency of PSII (*F*_q_'/*F*_m_') was measured (green) every hour of a measurement day over two growing seasons. Examples of a single measurement run at 12.00 h of the two glasshouse trials are shown (*n*=688 respective 700 measurements). (C) The *F*_q_'/*F*_m_' was measured by hand-held MultispeQ devices in the field on 14 d in three growing seasons. (D) Examples of a single measurement day of the three field trials are shown (*n*=1879, 1359, and 1203 measurements, respectively). (E) In total, 178 climbing bean lines were phenotyped and partially replicated in the five trials. The two glasshouse trials (CKA20D and CKA20E) were carried out at Campus Klein-Altendorf in 2020 in Germany whereas the three field trials (Dar18B, Dar19B, and Pal19D) took place in Darién (Dar) and Palmira (Pal) in Colombia in 2018 and 2019. The number of lines for each trial is given in parentheses. (F) Probability density diagram of the acquired *F*_q_'/*F*_m_' and its associated photosynthetic photon fluence rate (PPFR) and temperature values for the five trials (*n*=77 780 measurements).

Plants were grown in 2 liter black pots (square opening 12 × 2 cm) in an unheated glasshouse located at the agricultural research station Campus Klein-Altendorf of the University of Bonn, Germany (50°36'50.7''N, 6°59'38.9''E, altitude of 185 m a.s.l.). The trials, CKA20D and CKA20E, were sown on the first of September and October in 2020, respectively. The pots were filled with ~2 liters of turf–clay substrate (ED73, Einheitserdewerke, Sinntal-Altengronau, Germany). The plants were grown in six rows with 1.5 m row spacing and 22 pots per row with 14 cm spacing (Supplementary Fig. S1). The first and last pot in a row were excluded as a border. No fertilizers or plant protection agents were used. Four seeds per pot were seeded and thinned after ~2 weeks in order to grow two plants per pot. Bamboo stakes (~1 m) were inserted into each pot as support for the climbing beans. Drip irrigation was used twice a week to ensure well-watered growth conditions. A total of 136 lines were evaluated in two glasshouse trials (CKA20D, *n*=86 and CKA20E, *n*=85 lines), in a partially replicated design with four check lines in six replicates (Fig. 1A, B). The PPFR and temperature were measured every minute by a LI-COR sensor (LI-COR, Inc., Lincoln, NE, USA) and a HMP110 temperature sensor (Vaisala, Helsinki, Finland), respectively, at ~0.5 m above the pots. No artificial heating or lighting was used in the glasshouse.

#### Field

Plants were grown in the field in Darién, Colombia (3°53'31''N, 76°31'00''W, altitude of 1491 m a.s.l.) and in Palmira (3°30'03.0''N, 76°21'03.5''W, altitude of 965 m a.s.l.) as described by Keller *et al.* (2022a). Briefly, the trials in Darién, Dar18B (*n*=120 lines) and Dar19B (*n*=126 lines), were sown in the second season of the year in 2018 and 2019, respectively. The trial in Palmira (Pal19D, *n*=117 lines) was sown in the fourth season of the year in 2019. The soil types were an Inceptisol and Mollisol in Darién and in Palmira, respectively. The plants were treated and irrigated according to the standard CIAT protocol and empirical knowledge of the field. The PPFR values were recorded by the MultispeQ during every measurement. A total of 127 lines were evaluated in the field in 1–3 replicates (Fig. 1C, D).

### Phenotyping

The light-induced fluorescence transient (LIFT) and the MultispeQ devices measured photosynthetic performance under incident sunlight in the glasshouse and field, respectively. Both devices measure the *F*_q_'/*F*_m_' by probing the ChlF signal. Additionally, the LIFT device measures the full reflectance spectrum on the measured target while the MultispeQ is limited to only several spectral bands. Finally, biomass and grain yield were measured by destructive phenotyping at harvest.

#### Light-induced fluorescence transient

The ChlF and spectra were measured using the LIFT-REM device (Soliense Inc., New York, NY, USA), mounted on an automated platform that scans the pots from a distance of 1.5 m in the glasshouse (Fig. 1A). Measurements were automated by scanning the plant canopy at a speed of 3 cm s^−1^, as described by Keller *et al.* (2019a). Briefly, ChlF measurements were acquired by using a fast repetition rate flash (FRRF) every 1.5 s. The FRRF generates ~40 000 µmol photons m^−2^ s^−1^ of blue light (445 nm) with an excitation circle of 7 cm^2^ at a distance of 60 cm. The focus was then adjusted and optimized to a measuring distance of 1.25 m with a dynamic range of ~20 cm, optimizing the measurements for leaves between 15 cm and 35 cm above the ground. The minimum ChlF yield was determined by the first flashlet, and the maximum ChlF yield (*F*_m_') was the average of the 301st and 302nd flashlet (Keller *et al.*, 2019b). The difference between the two ChlF yields represents the variable ChlF (*F*_q_'), which is used to calculated *F*_q_'/*F*_m_' (Baker, 2008). Spectral measurements were acquired between the FRRFs through the LIFT lens using the LIFT’s integrated STS-VIS spectrometer (Ocean Insight, Orlando, FL, USA). Wavelengths were recorded between 400 nm and 800 nm with a resolution of 0.46 nm and an acquisition time of 1 s. This resulted in ~3 measurements per pot and hour of a measurement day. The MERIS terrestrial chlorophyll index (MTCI) was calculated as:


$$\begin{array}{l} MTCI=\left(R754-R710\right)/\left(R710+R680\right) \end{array}$$ (1)

where R indicates the wavelength. The photochemical reflectance index (PRI) and normalized difference vegetation index (NDVI) were calculated as described in Keller *et al.* (2022b).

#### MultispeQ

The hand-held MultispeQ v.1 (PhotosynQ, USA) was used to acquire *F*_q_'/*F*_m_' in the field (Kuhlgert *et al.*, 2016). The adaxial leaf side of upper, fully irradiated, youngest, but already developed, leaves was measured in the early mornings (09.00 h–11.00 h) and early afternoons (13.00 h–15.00 h) to capture all possible phenotypic variability and to get information on genotype acclimation. Measurements were taken from the central part of the trifoliate, avoiding the central vein. The measured height was set at ~1.5 m, according to the growth stage and development of each line. The previous measurements showed that plant height from where the measurements were taken did not significantly affect the values (comparing fully developed leaves on the outer canopy; data not shown). The relative chlorophyll content was estimated by the transmitted red (650 nm) and infrared (940 nm) light using a series of measurements under increasing light intensities (Markwell *et al.*, 1995; Kuhlgert *et al.*, 2016). The spectral bands were calibrated using color cards provided by the manufacturer. All three trials were measured using the Photosynthesis RIDES no open/close protocol (photosynq.org/protocols).

#### Grain yield and agronomic traits

Agronomic traits, especially grain yield, for the VEC lines are publicly available (Keller *et al.*, 2022a). To determine above-ground biomass grown in the glasshouse, plants per pot were cut at the soil level, dried at 75 °C for 48 h, and weighed.

### Data preparation

#### Grain yield and biomass

For the glasshouse trails, each biomass phenotype (*y*_*ijkl*_) was modeled with an effect for the experiment (*E*_*i*_), the line (*L*_*j*_), the G×E (*LE*_*ij*_), the row (row_*k*_), and the column (column_*l*_) of the pots:


$$\begin{array}{l} y_{ijkl}=E_{i}+L_{j}+LE_{ij}+row_{k}+column_{l}+{ɛ}_{ijkl} \end{array}$$ (2)

The adjusted means for biomass were then extracted over both trials as well as for each separate trial, for each breeding line.

Regarding the three field trials, the spatially corrected adjusted means for grain yield and seed iron concentration (SdFe) overall and for the field trials separately were taken from Keller *et al.* (2022a).

#### Operating efficiency of PSII (*F*_q_'/*F*_m_')

Measurements under low light conditions (*>*100 µmol photons m^−2^ s^−1^) and, for the LIFT, with a signal-to-noise ratio <30 were discarded. All variables were cleaned for outliers which were more distant from the median than 2.5 times the interquartile range. The LIFT measurements were associated with the corresponding pot (breeding line) according to the position of the platform recorded every 30 cm (Fig. 1). The measurements were then associated with the corresponding PPFR value recorded in the same minute.

Regarding the field trials, the MultispeQ device was used to measure *F*_q_'/*F*_m_' and the environmental variables.

### Modeling of *F*_q_'/*F*_m_'

#### Basic line model

Measured *F*_q_'/*F*_m_' values (*y*_*jm*_) were modeled in each experiment separately using fixed effects for each breeding line *j* (*L*_*j*_), for the square root of PPFR at time point *m* (*P*_*m*_), and for the interaction between line and the square root of PPFR (*LP*_*jm*_), as well as an error term (ε_*jm*_):


$$\begin{array}{l} y_{jm}=\mu +L_{j}+P_{m}+LP_{jm}+{ɛ}_{jm} \end{array}$$ (3)

For the field trials, *L*_*j*_ was replaced by a plot effect to account for spatial variability in the field (even between the same lines).

#### Line model with interaction for the glasshouse and field

To obtain adjusted means and responses for the glasshouse and field, model (3) included a fixed effect for each experiment (*E*_*i*_) and an interaction between experiment, line, and PPFR at time point *m* (*ELP*_*ijm*_). To account for the absorbance of the incoming light, an effect was added for the spectral index MTCI and the relative chlorophyll content (*A*_*m*_) at the time point *m* for the LIFT and MultispeQ device, respectively. To account for light heterogeneity at the top of the canopy, the reflectance (*B*_*m*_) at time point *m* was used to model *F*_q_'/*F*_m_' derived from LIFT canopy scans in the glasshouse. In the field, the *F*_q_'/*F*_m_' values measured at the leaf level were corrected for an effect for each of the MultispeQ devices. This resulted in the following model for *F*_q_'/*F*_m_' values (*y*_*ijm*_) with the associated error term (ε_*ijm*_):


$$\begin{array}{l} y_{ijm}=\mu +E_{i}+L_{j}+P_{m}+ELP_{ijm}+A_{m}+B_{m}+{ɛ}_{ijm} \end{array}$$ (4)

Adjusted means and responses for *F*_q_'/*F*_m_' were separately calculated for the glasshouse and the field experiments. *F*_q_'/*F*_m_' data points were classified as measurement error and discarded when they were a Cook’s distance farther from the median than 50 times the upper quartile range. Breeding lines with <30 available measurements were discarded from the analysis to ensure robust regression results. Previously, the sum of squares for each variable was calculated by adding the explanatory variables PRI, NDVI, relative chlorophyll content, and air temperature as additive effects for each line at time point m, as: $C_{m}=\sum_{q=1}^{Q}c_{mq}*\gamma_{q}$, where Q is the number of additional explanatory variables, *c*_*mq*_ is the measured value of the *q*th variable at time point *m*, and γ_*q*_ is the main effect (coefficient) for each variable. This extension of model (4) resulted in:


$$\begin{array}{l} y_{ijm}=\mu +E_{i}+L_{j}+P_{m}+ELP_{ijm}+A_{m}+B_{m}+C_{m}+{ɛ}_{ijm} \end{array}$$ (5)

The variables constituting *C*_*m*_ were dropped in model (4) because they explained <1.5% of the variance.

#### Response to PPFR

From models (3) and (4), the adjusted genotypic means for *F*_q_'/*F*_m_' and the response of *F*_q_'/*F*_m_' to PPFR (Response_G:PPFR_) were extracted over all trials and separately for the glasshouse and field trials using the emmeans R package, as described by Keller *et al.* (2022b). Briefly, the Response_G:PPFR_ refers to the coefficients *b*_*ELP*_ corresponding to the *ELP* term as *ELP*={*ELP*_*ijm*_}=*b*_*ELP*_×*Z*_*LE*_×*X*_*P*_ where *Z*_*LE*_ is a design matrix for the lines in each experiment and *X*_*P*_ is a vector of the PPFR values. In other words, *b*_*ELP*_ describes the slope of the *F*_q_'/*F*_m_' values with increasing PPFR for each breeding line.

### Statistical analysis

#### Correlation analysis

Adjusted means for biomass and grain yield were correlated to the Response_G:PPFR_ of each breeding line separately for the glasshouse and the field trials based on model (4). Pearson correlation coefficient (*r*) was then calculated.

#### Post-hoc significance analysis

The Tukey HSD test was used to perform post-hoc significance analysis between the lines in the upper and lower 20% percentiles of the Response_G:PPFR_ as well as the lines in between (intermediate), forming three different groups in terms of photosynthetic energy conversion efficiency.

#### Heritability calculation

Heritability (*H*^2^) of the derived Response_G:PPFR_ was calculated between the different experiments based on model (4). Heritability of the biomass was calculated based on model (2). In both cases, the following equation was used:


$$\begin{array}{l} H^{2}=\frac{\sigma L}{\left(\sigma L+\sigma_{\;ɛ}\sqrt{n}\right)} \end{array}$$ (6)

where σ_*L*_ was the variance of the breeding lines, and σ_ε_ the error variance, which was divided by the averaged number of replicates ($\bar{n}$) to account for the unbalanced design.

### Genomic predictions

The GEBV for biomass and grain yield were calculated for three different models using the Bayesian generalized linear regression (BGLR) R package (Pérez and de los Campos, 2014). A Gaussian prior was assumed for the random effects. The Gibbs sampler generated 16 000 iterations of which the first 4000 were burned-in and the remainder were thinned by factor 5. Genomic information for 14 913 single nucleotide polymorphism (SNP) markers was already available for the climbing bean lines (Keller *et al.*, 2022a).

#### Basic genotype model

In the genotype model, the phenotype (*y*_*ij*_) was described as the sum of a fixed effect for the *i*th experiment (*E*_*i*_), a random effect for the *j*th genotype (*g*_*j*_) and the error ε_*ij*_:


$$\begin{array}{l} y_{ij}=E_{i}+g_{j}+{ɛ}_{ij} \end{array}$$ (7)

where *g ~N*(0*,K*σ_*g*_^2^) with K as the kinship matrix. The kinship matrix based on the SNP matrix was calculated using the rrBLUP package.

#### G×E model

In the G×E model, an interaction effect between the *i*th location and the *j*th genotype (*gE*_*ij*_) was added:


$$\begin{array}{l} y_{ij}=E_{i}+g_{j}+gE_{ij}+{ɛ}_{ij} \end{array}$$ (8)

where *gE ~N*(0,I ⊗ *K*σ_*gE*_^2^) with I as the identity matrix for the experiments and ⊗ denotes the Kronecker product.

#### G×Ec model

In the G×Ec model, the location effect was replaced (parameterized) by measured values, namely the Response_G:PPFR_ (*v*_*i*_), in the *i*th environment. Additionally, an interaction effect between the *i*th environmental covariates—in this case the physiological covariates (*V*=*vv*^ʹ^)—and the *j*th genotype (*gV*_*ij*_) was added:


$$\begin{array}{l} y_{ij}=E_{i}+v_{j}+g_{ij}+gV_{ij}+{ɛ}_{ij} \end{array}$$ (9)

with *v ~N*(0,*V* σ_*v*_^2^), *gV ~N*(0,*V* ⊗ *K*σ_*g*_^2^), and ε *~N*(0,Iσ_ε_^2^).

#### Cross-validation

Cross-validation was carried out to predict new lines in an observed environment (CV1) and new lines in a new environment (CV2). CV1 gives information about the prediction accuracy within one trial while CV2 represents a more practical situation where new lines are predicted for a new season (i.e. environment). CV1 and CV2 were calculated for all three models presented. In the case of model (9), the ChlF data would be known for all lines; that is the Response_G:PPFR_ would also be available for new lines and in a new environment. The cross-validation was 60-fold where the dataset was randomly divided into three parts 10 times and each part was validated based on the training of the remaining two-thirds of the dataset.

### Genome-wide association study

GWAS was carried out using the Bayesian-information and Linkage-disequilibrium Iteratively Nested Keyway (BLINK) algorithm from GAPIT (Huang *et al.*, 2019). The first three principal components of the SNP matrix were used to correct for population structure as described for this climbing bean panel in Keller *et al.* (2022a). SNP markers with a minor allele frequency <5% were excluded. The effect of significant markers was visualized using boxplots for the trials of the current study and for six previous trials carried out by Keller *et al.* (2022a) and two trials by Barbosa *et al.* (2018). Candidate genes were identified within a region with a maximal distance of 100 000 bp from an identified significant marker–trait association and based on their relationship to the photosynthetic apparatus using gene annotations from Phytozome v13 (Goodstein *et al.*, 2012).

### Data visualization

Plots were done in R using ggplot2 with ggh4x extension (Wickham, 2016). The magick R package was used for images (Ooms, 2023).

## Results

The *F*_q_'/*F*_m_' of climbing bean lines was assessed throughout the growing season by taking ~700 measurements per hourly scan in the glasshouse (Fig. 1A, B) and ~1500 measurements per day in the field (including morning and afternoon measurements; Fig. 1C, D). Between 80 and 126 lines per trial were phenotyped, resulting in 178 lines of which 26 were measured in all five trials (Fig. 1E). A total of 79 645 measurements were acquired over the growing seasons in 57 d under fluctuating conditions (Fig. 1F). The associated weather data represented the growing conditions, which varied across the five trials. PPFR and temperature in particular varied between the glasshouse and field trials. The PPFR and temperature values were highest in the Palmira field trial (Pal19D) and lowest in the CKA20E glasshouse trial. The PPFR explained 10.3% and 69.9% of the variance for *F*_q_'/*F*_m_' in the glasshouse and field trials using model (5), respectively (Tables 1, 2). Additionally, ~1% was explained by the square root of PPFR. Air temperature as well as the measurement dates accounted for <1% of the variation for *F*_q_'/*F*_m_'. Reflectance and MTCI explained 7.0% and 4.6% of the variation for *F*_q_'/*F*_m_', respectively, in the glasshouse canopy scans (Table 1), while relative chlorophyll content explained 1.7% in the field (Table 2). The breeding lines (i.e. the genotypes) explained 10.0% and 1.5% of the variance for *F*_q_'/*F*_m_' in the glasshouse and field trials, respectively.

**Table 1.** Explanatory variables of the operating efficiency of PSII (*F*_q_'/*F*_m_') in two climbing bean glasshouse trials

Explanatory variable	df	Sum of squares	Explained variance
			(%)
Trial	1	163.757	22.253
PPFR	1	75.915	10.316
Line	130	73.529	9.992
Reflectance	1	51.271	6.967
MTCI	1	33.618	4.568
Trial:Line	34	12.463	1.694
Trial:MTCI	1	11.428	1.553
sqrt(PPFR)	1	7.662	1.041
PPFR:Line	130	7.6	1.033
PRI	1	5.502	0.748
NDVI	1	5.268	0.716
Temperature	1	2.647	0.36
Date	1	2.504	0.34
MTCI:NDVI	1	2.455	0.334
Trial:MTCI:NDVI	1	0.868	0.118
Trial:PPFR:Line	34	0.57	0.077
Trial:NDVI	1	0.425	0.058
Trial:Reflectance	1	0.093	0.013
Trial:PPFR	1	0.024	0.003
Residuals	62 741	278.302	37.818

The degrees of freedom (df), associated sum of squares, explained variance, and the residual are calculated using linear regression according to model (5). The explanatory variables were trial, photosynthetic photon fluence rate (PPFR), line, MERIS terrestrial chlorophyll index (MTCI), normalized difference vegetation index (NDVI), photochemical reflectance index (PRI), reflectance, the square root (sqrt) of PPFR, temperature, and date, as well as the interactions between variables denoted by the ‘:’ symbol. In total, 66 550 data points of 131 breeding lines were acquired by automated chlorophyll fluorescence (ChlF) measurements.

**Table 2.** Explanatory variables of the operating efficiency of PSII (*F*_q_'/*F*_m_') in three climbing bean field trials

Explanatory variable	df	Sum of squares	Explained variance
			(%)
PPFR	1	54.525	69.917
Trial	2	3.019	3.872
Chlorophyll	1	1.314	1.686
Line	126	1.176	1.508
Trial:Line	234	0.851	1.091
Trial:Device.ID	13	0.845	1.083
Trial:PPFR:Line	234	0.685	0.879
Date	13	0.649	0.832
sqrt(PPFR)	1	0.563	0.722
PPFR:Line	126	0.49	0.629
Temperature	1	0.007	0.009
Trial:PPFR	2	0.001	0.001
Residuals	12 340	13.859	17.771

The degrees of freedom (df), associated sum of squares, explained variance, and the residual are calculated using linear regression according to model (5). The explanatory variables were photosynthetic photon fluence rate (PPFR), trial, line, relative chlorophyll content, date, and temperature, as well as the interactions between variables denoted by the ‘:’ symbol. In total, 13 095 data points of 127 breeding lines were acquired by hand-held chlorophyll fluorescence (ChlF) measurements.

### Light use efficiency and yield

**Fig. 2.**
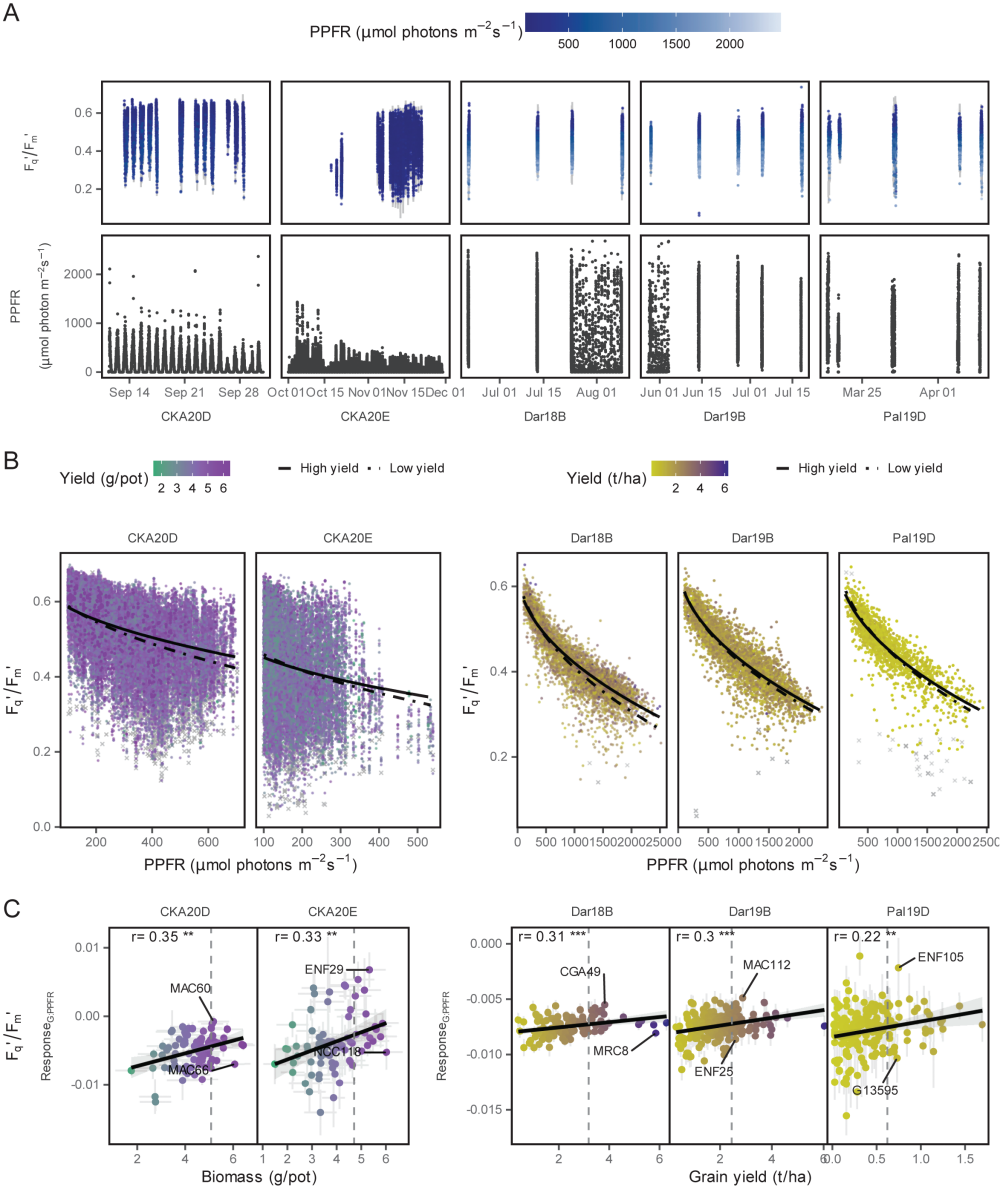
Dynamic photosynthesis is linked to biomass and yield. (A) Operating efficiency of PSII (*F*_q_'/*F*_m_') was measured under a fluctuating photosynthetic photon fluence rate (PPFR) over the season in five trials (*n*=72 453 selected in colors; discarded data points in gray; total *n*=77 780). (B) The *F*_q_'/*F*_m_' was related to PPFR modeled with a square root term for the 20% percentile of high-yielding (solid line) and low-yielding lines (dotted line). (C) The response of *F*_q_'/*F*_m_' to PPFR (Response_G:PPFR_) was modeled according to model (4) and correlated to biomass and yield. Light gray areas show the 95% confidence interval of the regression line. Contrasting breeding lines for Response_G:PPFR_ within the 20% percentile of high-yielding lines (dashed line) are labeled. Gray error bars show the SEM. The significance of the Pearson correlation coefficient (*r*) is indicated as ***P<0.001; **P<0.01. The two glasshouse trials (CKA20D and CKA20E) were carried out at Campus Klein-Altendorf in 2020 in Germany whereas the three field trials (Dar18B, Dar19B, and Pal19D) took place in Darién (Dar) and Palmira (Pal) in Colombia in 2018 and 2019.

After filtering, *F*_q_'/*F*_m_' was modeled using 61 252 and 11 201 ChlF measurements taken in the glasshouse and field, respectively (Fig. 2A). The general response of *F*_q_'/*F*_m_' to PPFR was more pronounced in low-yielding lines, indicating lower light use efficiency (LUE), especially at high PPFR (Fig. 2B). The Response_G:PPFR_, calculated according to the basic model (3), showed significant correlations with yield in four trials (Supplementary Fig. S2). This highlights the importance of the response of *F*_q_'/*F*_m_' to PPFR for biomass production, although the Response_G:PPFR_ (i.e. the Line:PPFR interaction) explained <1% of the variation for *F*_q_'/*F*_m_' (Tables 1, 2). Model (4) calculated the Response_G:PPFR_ with additional effects for chlorophyll content, reflectance, and device ID. This model showed slightly higher correlations between Response_G:PPFR_ and yield, ranging from *r*=0.22 to 0.35 (Fig. 2C). The diagnostic plots showed that model residuals were independent over time, had constant variance, and no significant deviation from a normal distribution, indicating reasonable model fits (Supplementary Fig. S3). In contrast to Response_G:PPFR_, the adjusted mean of *F*_q_'/*F*_m_' was not shown to be a reliable predictor of biomass (Supplementary Fig. S4). A significant effect on yield was observed when lines were grouped according to the calculated Response_G:PPFR_. The breeding lines in the lower 20% quantile of the Response_G:PPFR_, corresponding to a high ϵ_e_, showed significantly higher yield compared with the lines in the upper 20% quantile in four out of five trials (Supplementary Fig. S5). This result confirms that the trait Response_G:PPFR_ can be used as a selection target to identify lines with increased yield potential.

### Correlation of traits and heritability

**Fig. 3.**
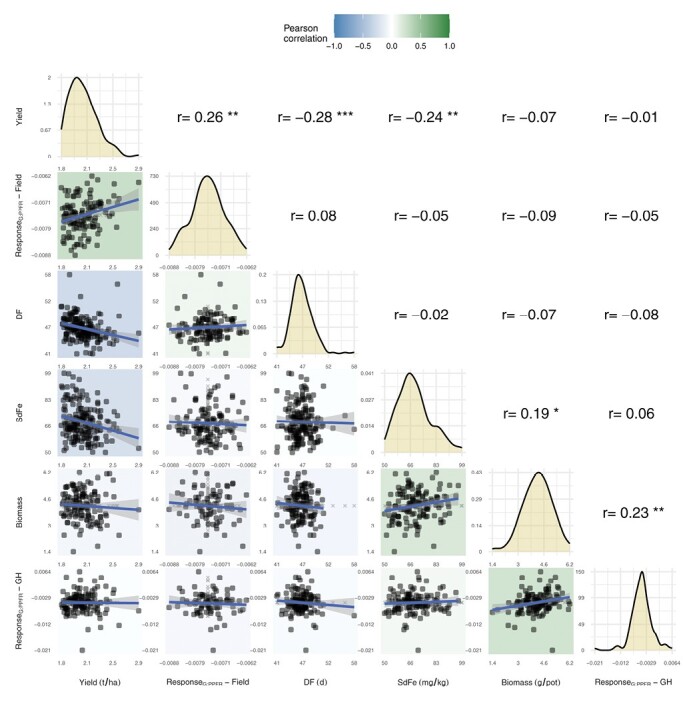
Correlations of overall adjusted means between photosynthetic and agronomic traits. The traits were grain yield, response of *F*_q_'/*F*_m_' to PPFR (Response_G:PPFR_) of the field trials, days to flowering (DF), seed iron concentration (SdFe), biomass, and Response_G:PPFR_ of the glasshouse (GH) trials. The diagonal shows a density diagram with the estimated distribution of the phenotypes (*n*=131 lines in the greenhouse and *n*=127 lines in the field; total n=178 lines). The significance of the Pearson correlation coefficient (*r*) from pairwise observations (with non-missing values) is indicated as ***P<0.001; **P<0.01; *P<0.05. Missing values were imputed using the population mean (shown in gray) and were only used to display complete pairwise observations.

The Response_G:PPFR_ values of the three field trials were positively correlated (Supplementary Fig. S6). An analysis of this trait across the field trials resulted in a heritability of 0.21 (Supplementary Table S1). In contrast, the Response_G:PPFR_ values of the two glasshouse trials were negatively correlated and, therefore, had a heritability of 0.15. These results indicate strong G×E effects, reflecting different growing conditions. The heritabilities of biomass in the glasshouse and grain yield in the field were higher, 0.36 and 0.53, respectively. The overall Response_G:PPFR_ between the three field trials and the two glasshouse trials was uncorrelated, as was biomass to grain yield (Fig. 3). However, both the overall Response_G:PPFR_ of the glasshouse and field trials were significantly correlated with biomass and grain yield (*r*=0.23 and 0.26), respectively, demonstrating prediction stability under similar growth conditions. Interestingly, above-ground biomass from the glasshouse trials correlated positively with SdFe evaluated in the field trials (*r*=0.19).

### Genomic assisted predictions

**Fig. 4.**
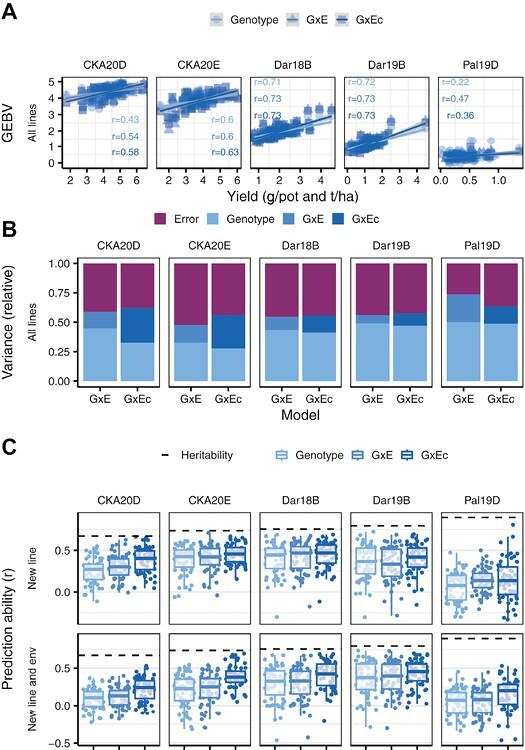
Predictions for biomass and grain yield in the two glasshouse and three field trials, respectively, using genomic and chlorophyll fluorescence (ChlF) data. (A) The genomic estimated breeding values (GEBVs) were calculated based on genotypic data, genotype by environment interaction (G×E), and interaction between genotype and the covariate (G×Ec) as described in models (7) to (9). The response of *F*_q_'/*F*_m_' to PPFR (Response_G:PPFR_) was used as covariate. The Pearson correlation coefficient (*r*) between measured and predicted value represents the prediction accuracy either of new lines in known environments or new lines in new environments. The two glasshouse trials (CKA20D, *n*=86 and CKA20E, *n*=80) were carried out at Campus Klein-Altendorf in 2020 in Germany, whereas the three field trials (Dar18B, *n*=120; Dar19B, *n*=126; and Pal19D, *n*=117) took place in Darién (Dar) and Palmira (Pal) in Colombia in 2018 and 2019. (B) The variance components for each model are shown in relative size. (C) Boxplot for biomass and yield prediction accuracy using two different 60-fold cross-validation scenarios and the three different models. The dashed line shows the square root of the spatial corrected heritability.

The genomic predictions of biomass and grain yield according to models (7) to (9) resulted in different prediction accuracies. The interaction terms in the G×E and G×Ec models, generally increased the prediction accuracy in the five trials (Fig. 4A). The variance explained by the genotype [i.e. the molecular markers as described in the basic genotype model (7)] ranged from 26.4% to 50.6% (Fig. 4B). Comparing the variance components of the G×E model (8) and the G×Ec model (9), the effects of the interaction term were similar (Fig. 4B). This result indicates that the Response_G:PPFR_ as covariate was almost sufficient to describe all the G×E effects on the yield. With early-stage phenotypic information available (the Response_G:PPFR_ in our study), the G×E model (8) performed best when predicting new lines in an observed environment (CV1) and new lines in a new environment (CV2) (Fig. 4C). The G×Ec model (9) achieved averaged prediction accuracies between 0.35 and 0.38, an increase in CV2 of 53.1% compared with the G×E model (8). When predicting new lines in a new environment (CV2), the prediction accuracy of the G×E model (8) was similar to the basic genotype model (7), indicating strong year-to-year effects. Using only genomic data, the highest accuracies were obtained in both Darién trials. However, only one of these trials (Dar19B) exceeded previous phenomic prediction accuracy (without any genomic data, Fig. 2).

### Genome-wide association study for photosynthetic response

**Fig. 5.**
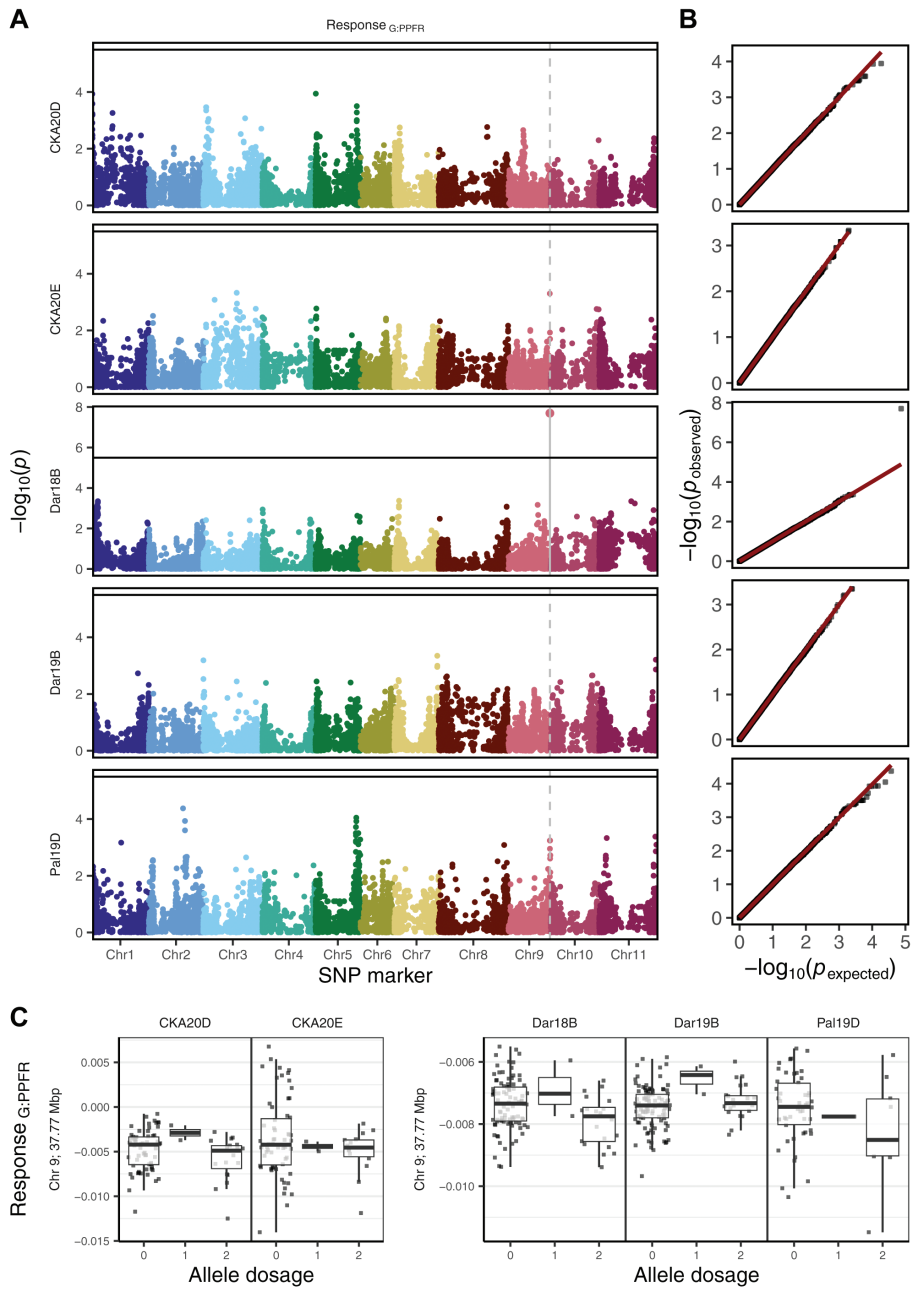
The genome-wide association study (GWAS) for response of *F*_q_'/*F*_m_' to PPFR (Response_G:PPFR_) of the five trials. (A) Manhattan plots showing the log-transformed *P*-value for every marker–trait association. The SNP markers are displayed according to their physical position on the genome. The Bonferroni-corrected significance level is indicated at 5% with a solid line. (B) The quantile–quantile plot of expected and observed *P-*values for every marker. (C) Boxplot of Response_G:PPFR_ according to allele dosage (0, 1, or 2 alternative alleles) of the significantly associated marker (Chr09_37766289_13052) for all five trials. The two glasshouse trials (CKA20D, *n*=86; and CKA20E, *n*=80) were carried out at Campus Klein-Altendorf in 2020 in Germany whereas the three field trials (Dar18B, *n*=120; Dar19B, *n*=126; and Pal19D, *n*=117) took place in Darién (Dar) and Palmira (Pal) in Colombia in 2018 and 2019.

Investigating genetic control over photosynthesis, GWAS revealed a significant association between Response_G:PPFR_ and the SNP marker (Chr09_37766289_13052) on Chr 9 at 37.77 Mbp in the Dar18B trial (Fig. 5A, B). Although not significant in the remaining trials, the effect of this marker was also observed in the CKA20D glasshouse and the Pal19D field trials (Fig. 5C). Furthermore, this marker caused pleiotropic effects on yield and SdFe in the complete VEC population including 290 lines evaluated in eight and four trials (Barbosa *et al.*, 2018; Keller *et al.*, 2022a), respectively (Supplementary Fig. S7). However, the marker was significant neither for yield nor for SdFe (Supplementary Fig. S8), suggesting that the Response_G:PPFR_ was a rather stable physiological component of those more complex traits. In fact, this marker (Chr09_37766289_13052) showed a positive effect on yield in six out of the eight field trials. That was more stable than several markers for yield identified in these trials (Supplementary Fig. S9). A candidate gene (Phvul.009G258500) expressing a curvature thylakoid 1 protein was identified in proximity at 37.84 Mbp, which could be responsible for the improved photosynthetic response.

## Discussion

Photosynthesis is a promising target for improving crop production, because the energy conversion efficiency of sunlight to biomass continues to show high genetic variation even in high-yielding breeding lines (Zhu *et al.*, 2010; Driever *et al.*, 2014; Keller *et al.*, 2022b). Our study highlights the potential of automated high-throughput ChlF measurements for the selection of thousands of breeding lines in early generations. This approach is valid in the new environments and when there is a possibility to use similar high-throughput techniques, especially where genomic selection is still very expensive. The positive relationship between Response_G:PPFR_ and biomass production was shown using single pot experiments (potentially single plants; we used two plants per pot) in the glasshouse. The data trends were confirmed with hand-held instruments in field conditions.

### Solar energy conversion and yield

The relationship between *F*_q_'/*F*_m_' and PPFR tended towards a linear response, a known result in field plants grown under fluctuating light conditions, and its slope was a robust yield predictor (Meacham *et al.*, 2017; Vialet-Chabrand *et al.*, 2017; Keller *et al.*, 2022b). Breeding lines which tolerate or benefit from high light intensities—showing a low Response_G:PPFR_ and a high ϵ_e_—have potential for high LUE and high yield (Keller *et al.*, 2022b). Identified lines with low Response_G:PPFR_ values and high yield (Fig. 2C) indicated (i) high transduction efficiency of photochemical energy to biomass, which may result from high root and stem conductivity and efficient carbon and mineral partitioning and (ii) the potential for higher yields of those lines when improving photosynthetic efficiency (Driever *et al.*, 2014). In the climbing bean lines studied, additional variation for symbiotic nitrogen fixation was reported by Barbosa *et al.* (2018), which may also contribute to differences in ϵ_e_. In grain legumes, the costs of nitrogen fixation increase sink strength, emphasizing conversion efficiency as a breeding target. These findings highlight the potential of the source–sink ratio as well as photosynthesis as breeding targets (Evans and Clarke, 2019; Wu *et al.*, 2023).

### Canopy and leaf photosynthesis

All trials and conditions in the current study showed a positive relationship between plant photosynthetic performance and yield. This result confirmed that Response_G:PPFR_, as suggested by Keller *et al.* (2022b), is a robust yield indicator, regardless of whether the bean leaves were measured from the top of the canopy via automated measurements, or directly at the leaf level via hand-held devices. However, the residuals were much higher in the glasshouse at the canopy level (37.8%, Table 1) than in the field at the leaf level (17.8%, Table 2). This finding probably represents the very different conditions in the field and glasshouse, where the lower PPFR affected the distribution of the residuals. It is likely that the spectral indices in the presented model (4) accounted partially for canopy structure and differences in leaf angles rather than in leaf pigment composition (Keller *et al.*, 2019a). Relative chlorophyll content showed a minor influence on *F*_q_'/*F*_m_', because it determines light absorption at the leaf level and further distribution into the canopy (Acebron *et al.*, 2023).

The top leaves of the canopy are the most important part to measure because ~70% of solar energy is absorbed by the outer canopy (Song *et al.*, 2013). In soybean, Roth *et al.* (2022) showed that varieties with early canopy closure have higher yield, highlighting the importance of high light interception efficiency. However, quantifying the influence of the inner canopy structure, in terms of photosynthesis and nutrient re-assimilation, may further improve overall phenotyping precision and generate deeper knowledge on the effect of photosynthesis–yield relationship.

### Photosynthesis and yield

Lopez *et al.* (2019) reported correlations between photosynthesis and grain yield of up to 0.8 in soybean. In wheat, Gutiérrez-Rodríguez *et al.* (2000) and Fischer *et al.* (1998) reported correlations of up to 0.79 between wheat yield and *g*_s_ when measured under light-saturated conditions (between 1200 µmol m^−2^ s^−1^ and 2000 µmol m^−2^ s^−1^). Our correlations were much lower, up to 0.43. The main reason for this difference is that we derived ChlF differently. Our method measured ET, which is subject to greater losses for biomass production than CO_2_ assimilation, which occurs in between these two processes. Importantly, this means that when breeding lines are selected using ChlF, both high transduction efficiency and low photorespiration are probably included as ‘hidden’ selection traits. The lower prediction accuracy in our study using ChlF is sufficiently compensated by the possibility of automation, which allows significantly higher throughput than gasometric measurements. Additionally, the difference in measurements between ET and CO_2_ assimilation facilitates selection of lines with less photorespiration. However, this point needs to be verified by testing the hypothesis that photorespiration is genetically diverse and varies in different environments, as recently reported for wheat lines (Coast *et al.*, 2019, 2021). Nevertheless, the current study showed a significant increase in yield when selecting for low Response_G:PPFR_, namely high ϵ_e_ and LUE (Supplementary Fig. S5). Overall, the robust correlations between photosynthetic performance and yield are promising targets for increasing selection intensity in breeding programs.

### Genetic control of photosynthetic response

One of the field trials identified a significant marker–Response_G:PPFR_ association on chromosome 9 at 37.77 Mbp (Fig. 5). This association was also visible (although not significant) in another field trial as well as in one glasshouse trial. Our results suggest some common genetic control under these different conditions, despite the lack of phenotypic correlation for Response_G:PPFR_ between glasshouse and field conditions. Furthermore, the identified marker showed a positive effect on grain yield and a negative effect on SdFe in previous field trials described by Keller *et al.* (2022a), suggesting that SdFe was likely diluted by increased photosynthates (Supplementary Fig. S7). Importantly, the marker was not detected with GWAS for grain yield, indicating Response_G:PPFR_ as a distinct, but physiological and causal component of yield. The genomic region of the marker revealed a candidate gene (Phvul.009G258500 at 37.84 Mbp, a curvature thylakoid 1 protein) controlling the formation of chloroplasts (Sandoval-Ibáñez *et al.*, 2021). Interestingly, the *GmFtsH25* gene was recently identified in soybean, where it increases photosynthesis under high light intensities and alters chloroplast structure, allowing a denser stacking of the thylakoids (Wang *et al.*, 2023). Our quantitative trait locus (QTL), however, needs validation in further studies, especially to clarify under which conditions it is effective. In a much larger population including bush beans, Keller *et al.* (2022a) identified another QTL for days to flowering in close proximity (<60 000 bp away) to our photosynthetic QTL. On chromosome 9, at 25.29 Mbp, Leitão *et al.* (2021) reported a QTL for *g*_s_ under glasshouse conditions using 158 common bean accessions. In conclusion, the Response_G:PPFR_ probably contributed to yield as one of several physiological components which is rather stable across environments.

### Phenomic or genomic selection?

Genomic data were used to predict grain yield and agronomic traits in 178 climbing bean lines. Our prediction accuracy was lower than that reported by Keller *et al.* (2022a), most probably due to the smaller pool of climbing bean lines in the training set. In bush beans, Keller *et al.* (2020) showed that the training set needed >200 lines to achieve the highest genomic prediction accuracy in an elite breeding panel. Additionally, phenotypic data collected at early stages in early-generation breeding plots have been shown to improve yield prediction (Rutkoski *et al.*, 2016). The Response_G:PPFR_ used in our study showed potential to add information about the particular environment in which new lines are to be predicted. The low correlation between Response_G:PPFR_ across trials, resulting in low broad-sense heritability, underlined that photosynthesis generally reflects the specific growing conditions in each trial (Keller *et al.*, 2019a), which is useful information for site-specific predictions. In agreement with this statement, the Response_G:PPFR_ almost completely described different environments, comparing the variance components of the G×E model (8) and the G×Ec model (9). Furthermore, phenomic predictions using Response_G:PPFR_ achieved even better prediction accuracy than the genomic prediction model (8) for new environments, except for one of the Darién trials (compare Fig. 2C with Fig. 4C). Similarly, in soybean, phenomic prediction outperformed genomic predictions in terms of accuracy (Lopez *et al.*, 2019). Combining phenomic and genomic data, as in the G×Ec model (9), increased the prediction accuracy in general. However, having phenotypic information of lines available in the newly used environment is not a realistic CV2 scenario for any breeding program. Acquiring phenotypic information, however, is very useful when genotypic information is not yet available and yield is difficult to assess, as is the case for selection in early generation breeding lines.

### Conclusion

Photosynthetic performance has been shown to be a critical yield component. Using automated photosynthesis phenotyping, biomass was predicted by the Response_G:PPFR_ in >130 common bean breeding lines in single pots. The phenomic prediction method also proved to be effective in the field using hand-held devices. Compared with genomic prediction based on molecular markers, phenomic prediction was more accurate in four out of five trials. Importantly, this suggests that phenomic prediction can be used as an early selection target, for example in *F*_3_ or *F*_4_ individuals in progeny rows, to screen large numbers of plants. Since the phenotyping accounts for G×E, breeding programs can improve selection and better understand adaptation, especially in new environments. Similarly, the identified significant association between marker (Chr09_37766289_13052) and the Response_G:PPFR_ trait may be used for marker-assisted selection on a physiological yield component where markers directly linked to yield are not stable throughout the environments. In contrast, genomic prediction was more accurate in a known location, confirming its potential to select more advanced (or phenomically pre-selected) breeding lines based on training data from the same or a similar location.

## Supplementary data

The following supplementary data are available at *JXB* online.

Table S1. Heritability (*H*^2^) by trait and conditions.

Fig. S1. Climbing bean lines at 43 days after sowing.

Fig. S2. Adjusted means of operating efficiency of PSII (*F*_q_'/*F*_m_') and the response of *F*_q_'/*F*_m_' to PPFR (Response_G:PPFR_) were modeled according to the basic model (3) and correlated to biomass and yield.

Fig. S3. Diagnostic plots for the residuals from operating efficiency of PSII (*F*_q_'/*F*_m_') values using model (4) for all trials.

Fig. S4. Adjusted means of operating efficiency of PSII (*F*_q_'/*F*_m_') and the response of *F*_q_'/*F*_m_' to PPFR (Response_G:PPFR_) were modeled according to the basic model (4) and correlated to biomass and yield.

Fig. S5. Breeding lines grouped for the response of *F*_q_'/*F*_m_' to PPFR (Response_G:PPFR_) differ in yield in all five trials.

Fig. S6. Correlations of adjusted means between traits and trials. The traits were yield, biomass, and response of *F*_q_'/*F*_m_' to PPFR (Response_G:PPFR_) of the five trials.

Fig. S7. Allele dosage effect for days to flowering (DF), seed iron concentration (SdFe), and grain yield of the molecular marker (Chr09 37766289 13052) on chromosome 9 at 37.77 Mbp significantly linked to response of *F*_q_'/*F*_m_' to PPFR (Response_G:PPFR_).

Fig. S8. The genome-wide association study (GWAS) for biomass, grain yield, and seed iron concentration (SdFe) of all available climbing bean (VEC) trials.

Fig. S9. Allele dosage effect for response of *F*_q_'/*F*_m_' to PPFR (Response_G:PPFR_) and yield of significantly associated molecular marker identified in trials with the climbing bean population (VEC).

Dataset S1. Chlorophyll fluorescence (ChlF) data from the field trials acquired by the MultispeQ device.

Dataset S2. Chlorophyll fluorescence (ChlF) data from the glasshouse trials acquired the light-induced fluorescence transient (LIFT) device.

Dataset S3. Biomass data from the glasshouse trials.

erad416_suppl_Supplementary_Table_S1_Figures_S1-S9

erad416_suppl_Supplementary_Dataset_S1

erad416_suppl_Supplementary_Dataset_S2

erad416_suppl_Supplementary_Dataset_S3

## Data Availability

Grain yield and agronomic traits are available from Keller *et al.* (2022a). The ChlF and biomass data from the glasshouse are provided in Supplementary Datasets S1–S3. The MultispeQ data are also available on the PhotosynQ data base after creating an account (Darién 2018: https://photosynq.org/projects/climbers-in-darien-2018; Darién 2019: https://photosynq.org/projects/19-04-climbers-darien; Palmira 2019: https://photosynq.org/projects/19-03-climbers-palmira).

## Abbreviations:

*A*_max_: maximum photosynthetic rate
Response_G:PPFR_: response of F_q_'/F_m_' to PPFR
ϵ_e_: energy conversion efficiency of intercepted PPFR to photochemical energy
*g*_s_: stomatal conductance
ChlF: chlorophyll fluorescence
ET: electron transport
*F*_q_'/*F*_m_': operating efficiency of PSII
FRRF: fast repetition rate flash
G×E: genotype by environment interaction
GEBV: genomic estimated breeding values
G×Ec: interaction between genotype and the covariate
GWAS: genome-wide association study
LIFT: light-induced fluorescence transient
LUE: light use efficiency
MTCI: MERIS terrestrial chlorophyll index
NDVI: normalized difference vegetation index
NPQ: non-photochemical quenching
PPFR: photosynthetic photon fluence rate
PRI: photochemical reflectance index
SdFe: seed iron concentration.
