# Supplementary material for: Linking photosynthesis and yield reveals a strategy to improve light use efficiency in a climbing bean breeding population
Source: J Exp Bot. 2023 Oct 25;75(3):901–16. doi: 10.1093/jxb/erad416 (PMC10837016; doi:10.1093/jxb/erad416)
Supplement: erad416_suppl_Supplementary_Table_S1_Figures_S1-S9 [file erad416_suppl_supplementary_table_s1_figures_s1-s9.pdf]

Supplemental Materials

1 Supplemental tables

Supplementary Table 1: Heritability (H^2) by trait and conditions. The $Response_{G:PPFR}$, grain yield and biomass were assessed in two glasshouse trials and three field trials including a total of 178 climbing bean lines.

Trait	Conditions	H^2
Response	Field	0.213
Response	Glasshouse	0.152
Yield	Field	0.526
Biomass	Glasshouse	0.356

2 Supplemental figures

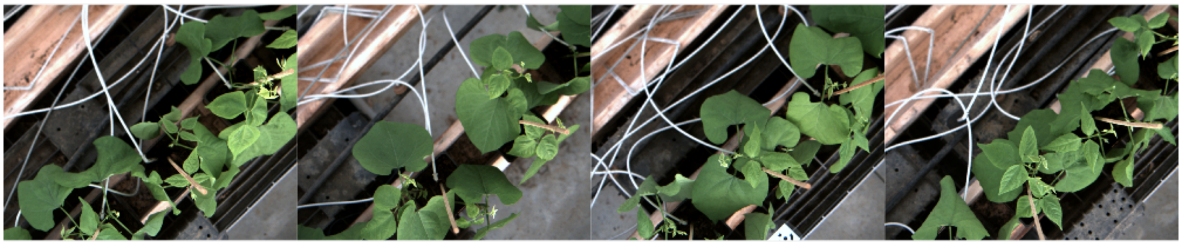

Supplementary Figure 1: Climbing bean lines at 43 days after sowing (DAS). These plants were grown in pots in the glasshouse at Campus Klein-Altendorf in the CKA20E trial in 2020.

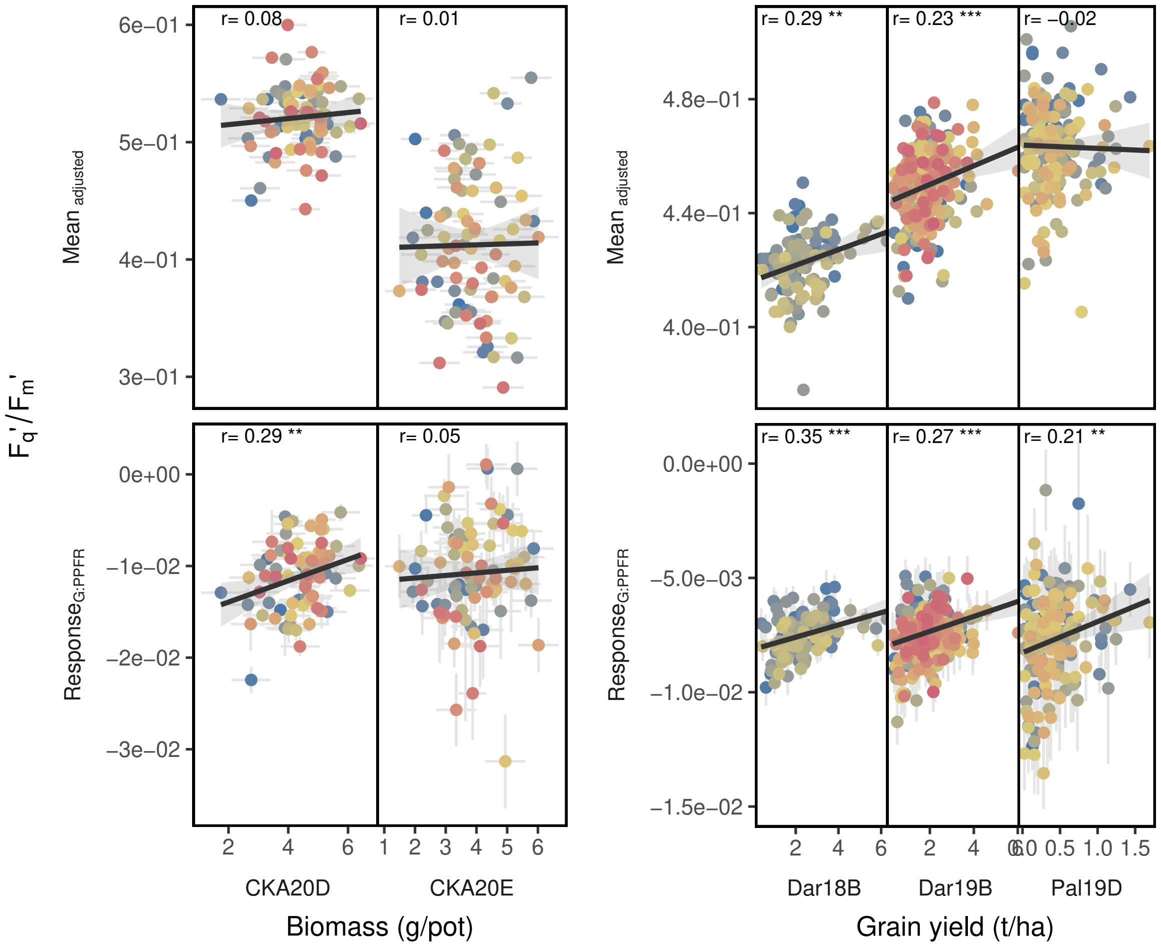

Supplementary Figure 2: Adjusted means of operating efficiency of photosystem II (F_q'/F_m') and the response of F_q'/F_m' to PPFR ($Response_{G:PPFR}$) were modeled according to the basic model (3) and correlated to biomass and yield. Light grey areas show the 95% confidence interval of the regression line. Different colors indicate the 178 breeding lines. The two glasshouse trials (CKA20D and CKA20E) were carried out at Campus Klein-Altendorf in 2020 in Germany whereas the three field trials (Dar18B, Dar19B and Pal19D) took place in Darién (Dar), Palmira (Pal) in Colombia in 2018 and 2019. The significance of the Pearson correlation coefficient (r) is indicated as '***': $p < 0.001$; '**': $p < 0.01$; '*': $p < 0.05$; '.': $p < 0.1$; ' ': not significant.

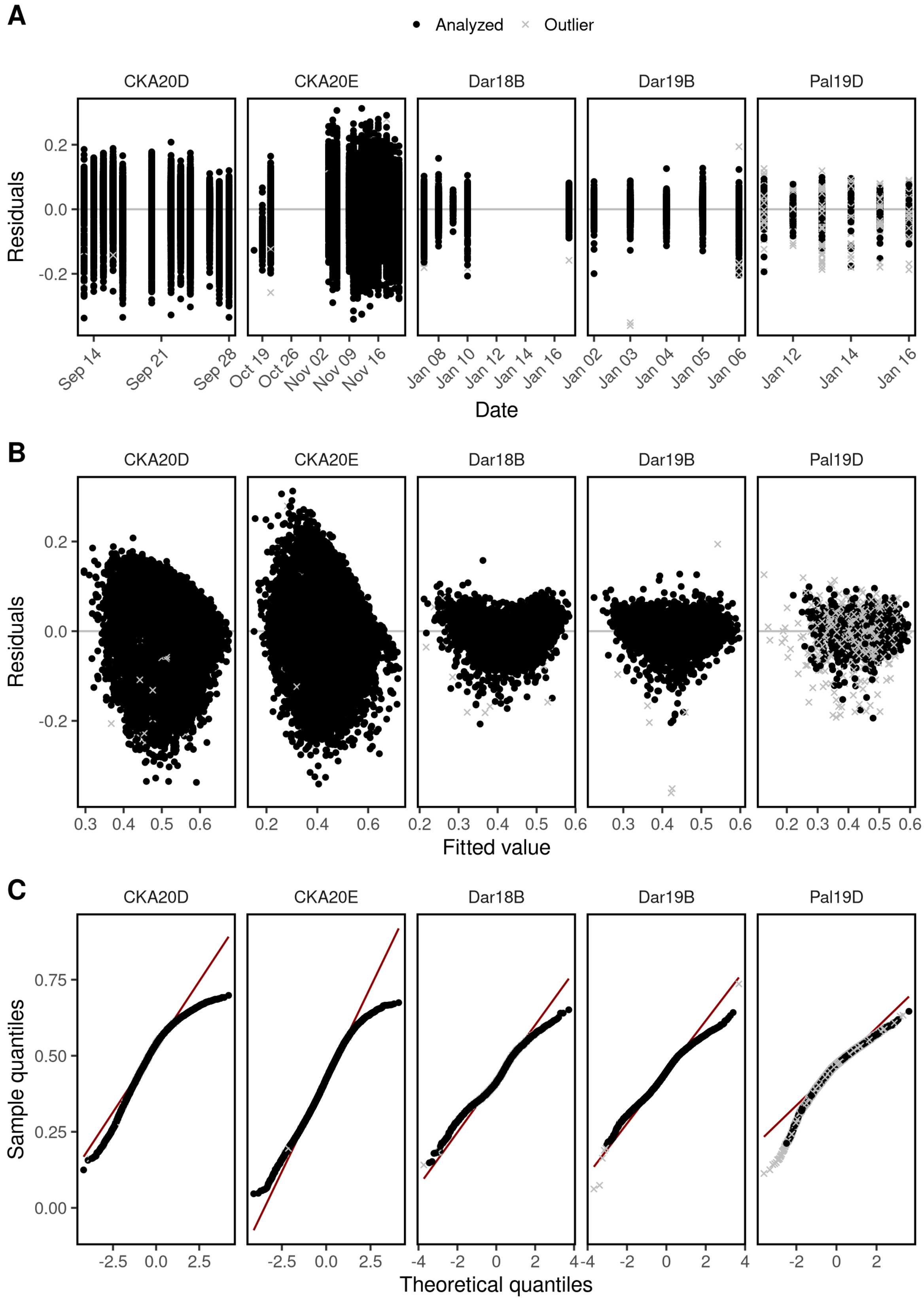

Supplementary Figure 3: Diagnostic plots for the residuals from operating efficiency of photosystem II (F_q'/F_m') values using model (4) for all trials. A: Residuals were plotted against date to check that residuals are independent. B: Residuals were plotted against fitted value to check for homoscedasticity. C: Sample quantiles were plotted against the theoretical quantiles to check for normal distribution of the residuals.

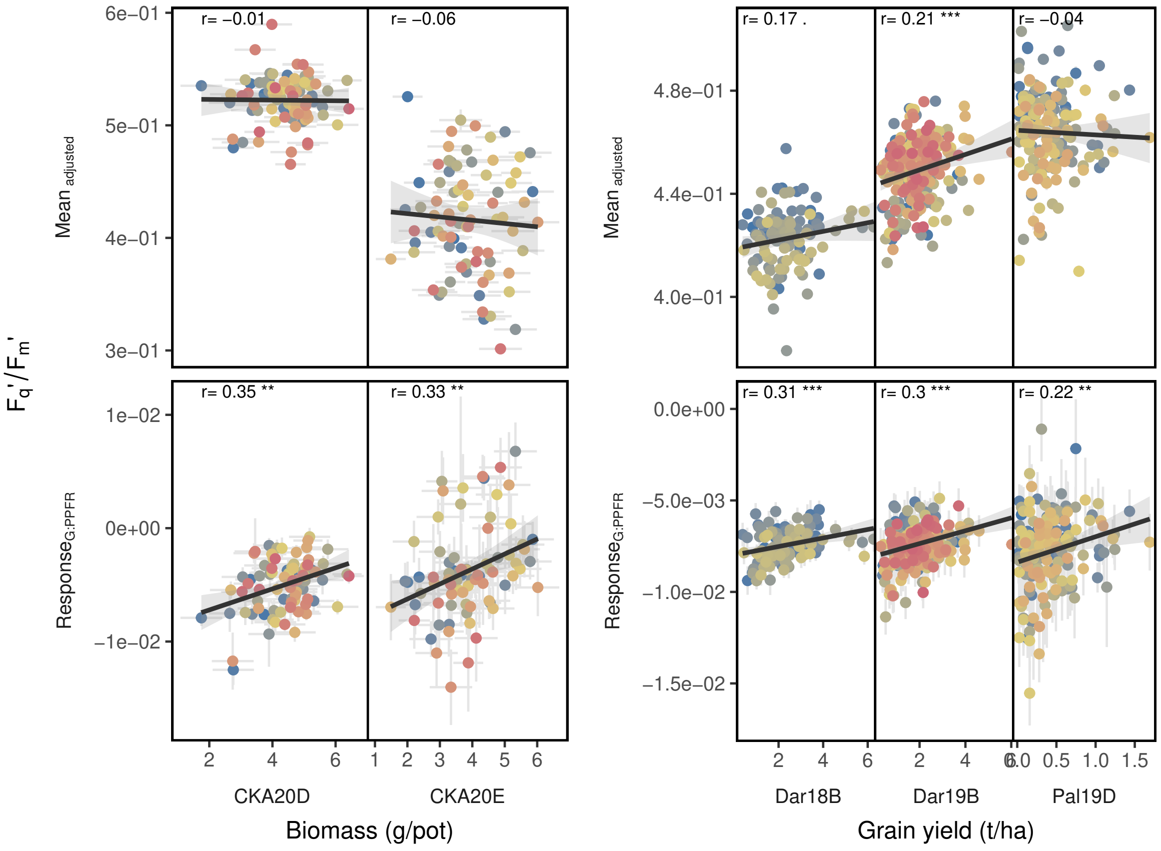

Supplementary Figure 4: Adjusted means of operating efficiency of photosystem II (F_q'/F_m') and the photosynthetic photon fluence rate (PPFR) in the glasshouse and field trials were modeled according to model (4) and correlated to biomass and yield. Light grey areas show the 95% confidence interval of the regression line. Different colors indicate the 178 breeding lines. The two glasshouse trials (CKA20D and CKA20E) were carried out at Campus Klein-Altendorf in 2020 in Germany whereas the three field trials (Dar18B, Dar19B and Pal19D) took place in Darién (Dar), Palmira (Pal) in Colombia in 2018 and 2019. The significance of the Pearson correlation coefficient (r) is indicated as '***': $p < 0.001$; '**': $p < 0.01$; '*': $p < 0.05$; '.': $p < 0.1$; ' ': not significant.

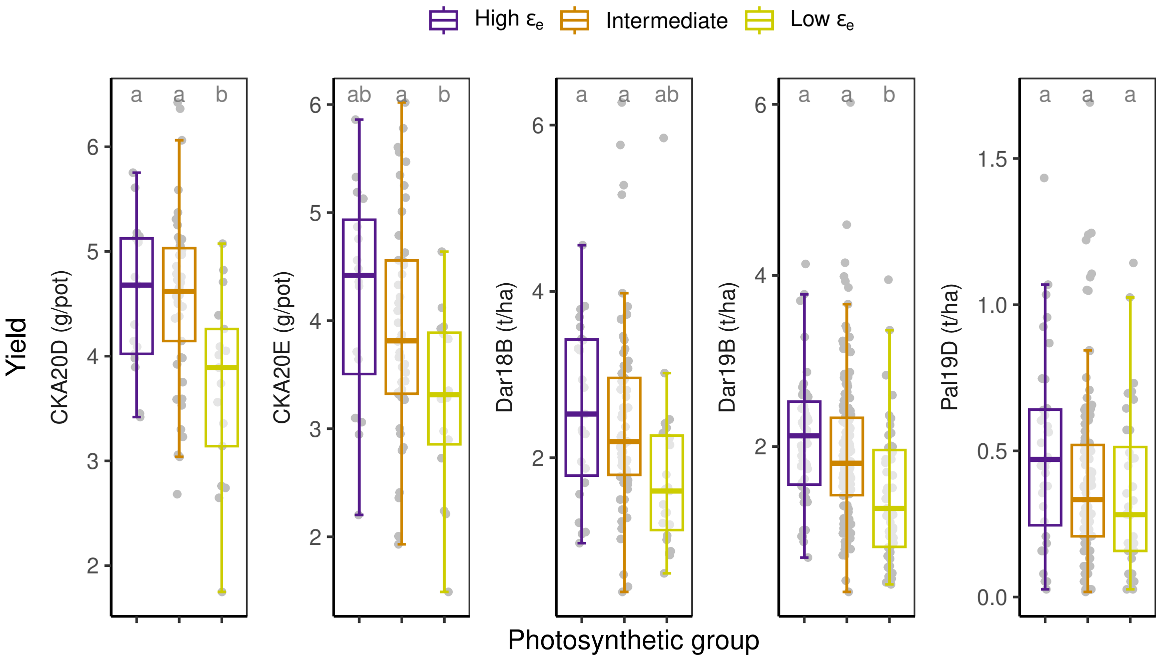

Supplementary Figure 5: Breeding lines grouped for the response of F_q'/F_m' to PPFR ($Response_{G:PPFR}$) differ in yield in all five trials. The boxplots for yield show lines of the lower and upper 20% percentile of the $Response_{G:PPFR}$, i.e., represent photosynthetic groups with low and high energy conversion efficiency of intercepted PPFR to photochemical energy (ϵ_c), as well as for the intermediate lines in-between. The two glasshouse trials (CKA20D and CKA20E) were carried out at Campus Klein-Altendorf in 2020 in Germany whereas the three field trials (Dar18B, Dar19B and Pal19D) took place in Darién (Dar), Palmira (Pal) in Colombia in 2018 and 2019. Same letters above the boxplots indicate that there is no significant difference on the 5% significance level according to Tukey HSD.

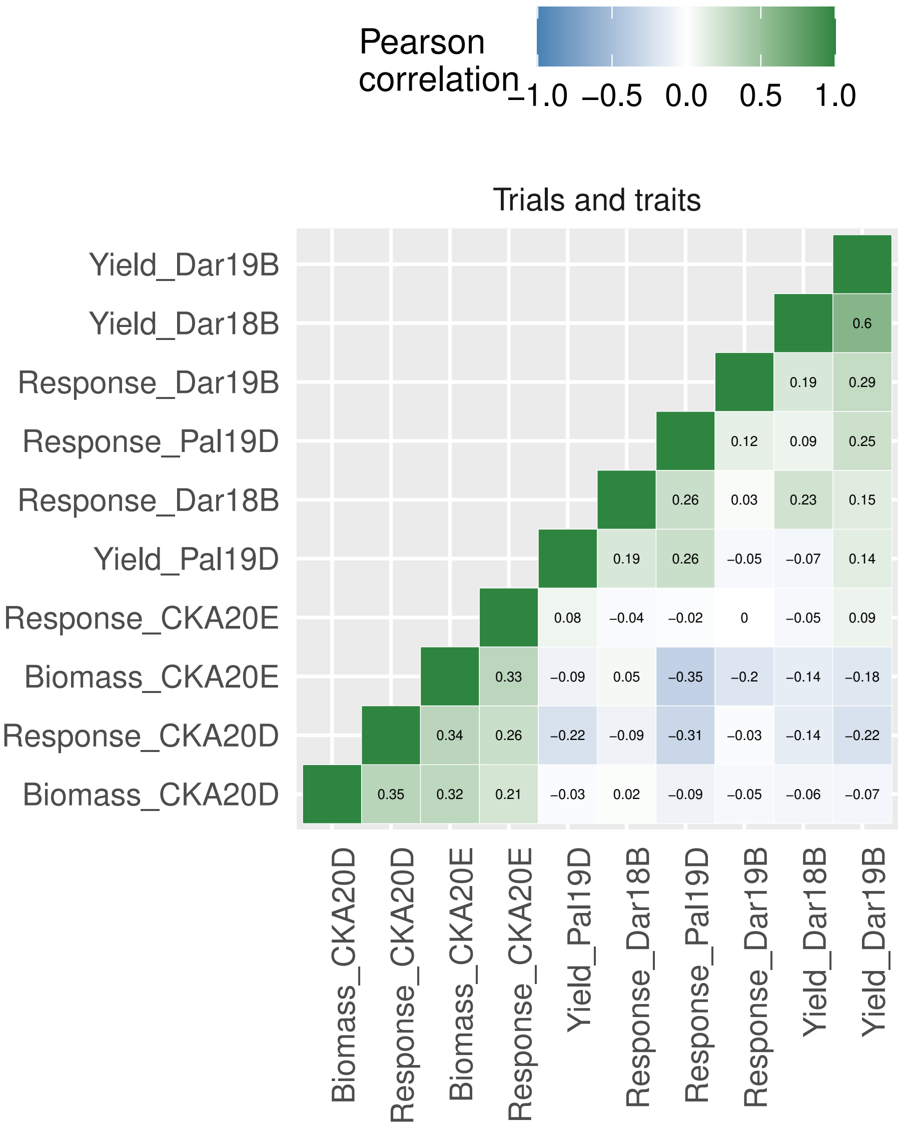

Supplementary Figure 6: Correlations of adjusted means between traits and trials. The traits were yield, biomass, response of F_q'/F_m' to PPFR ($Response_{G:PPFR}$) of the five trials. The two glasshouse trials (CKA20D and CKA20E) were carried out at Campus Klein-Altendorf in 2020 in Germany whereas the three field trials (Dar18B, Dar19B and Pal19D) took place in Darién (Dar), Palmira (Pal) in Colombia in 2018 and 2019.

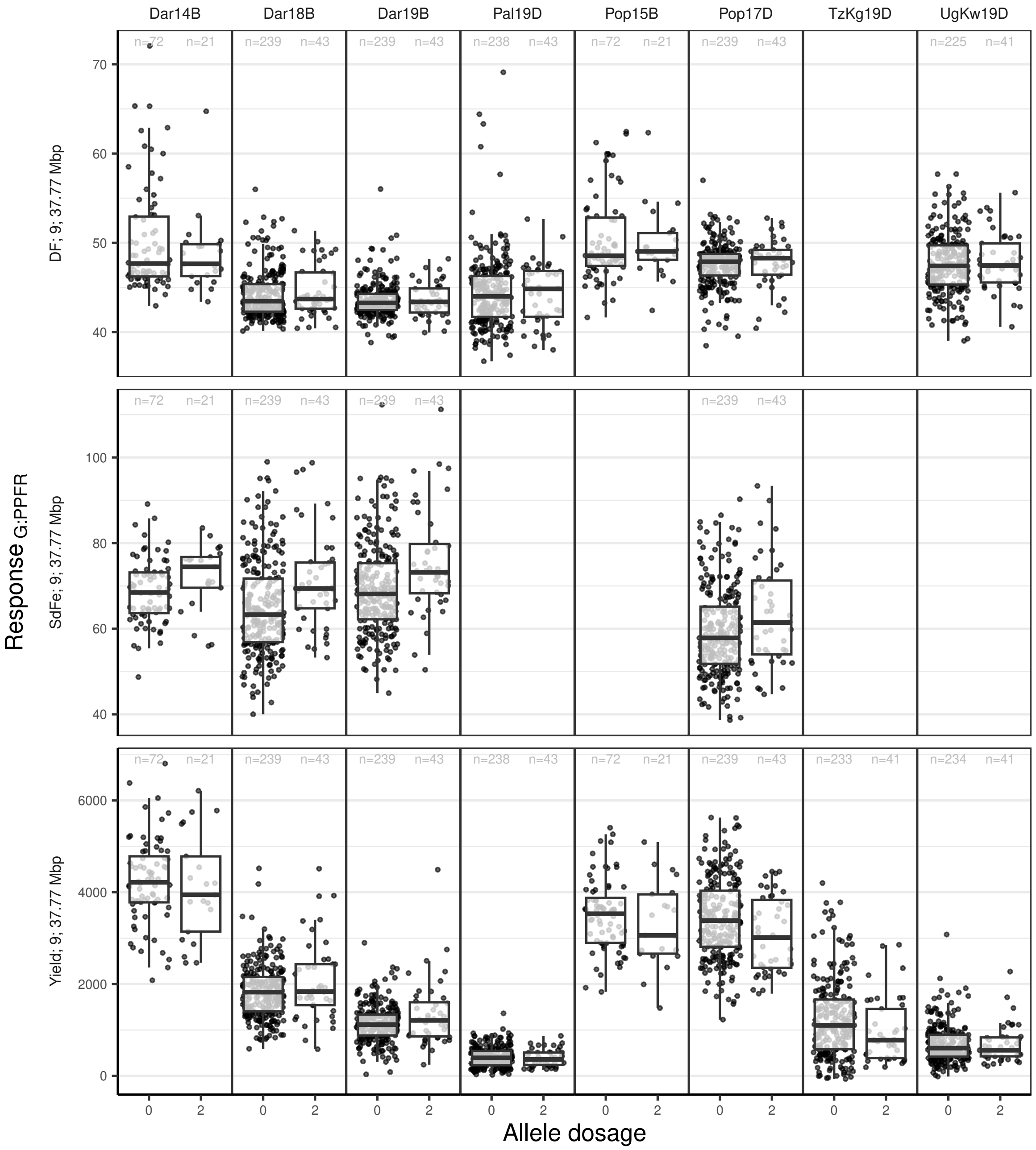

Supplementary Figure 7: Allele dosage effect for days to flowering (DF), seed iron concentration (SdFe) and grain yield of molecular marker (Chr09_37766289_13052) on chromosome 9 at 37.77 Mbp significantly linked to response of F_q'/F_m' to PPFR ($Response_{G:PPFR}$). The VEC population was previously phenotyped in eight field trials, of which six field trials were carried out with the complete population (n=290; Keller et al., 2022a) and two field trials included 43 additional climbing bean lines (Dar14A and Pop15B, n=93; Barbosa et al., 2018). Number of breeding lines at 0 or 2 allele dosage is indicated in grey. Breeding lines with homozygous loci (allele dosage=1) at specific marker position were excluded (n= 2 to 12) to highlight the homozygous effect. Trials were abbreviated based on the location Darién (Dar), Palmira (Pal), Popayán (Pop) in Colombia, Kagera in Tanzania (TzKg), or Kawanda in Uganda (UgKw), the year and the season (A to D).

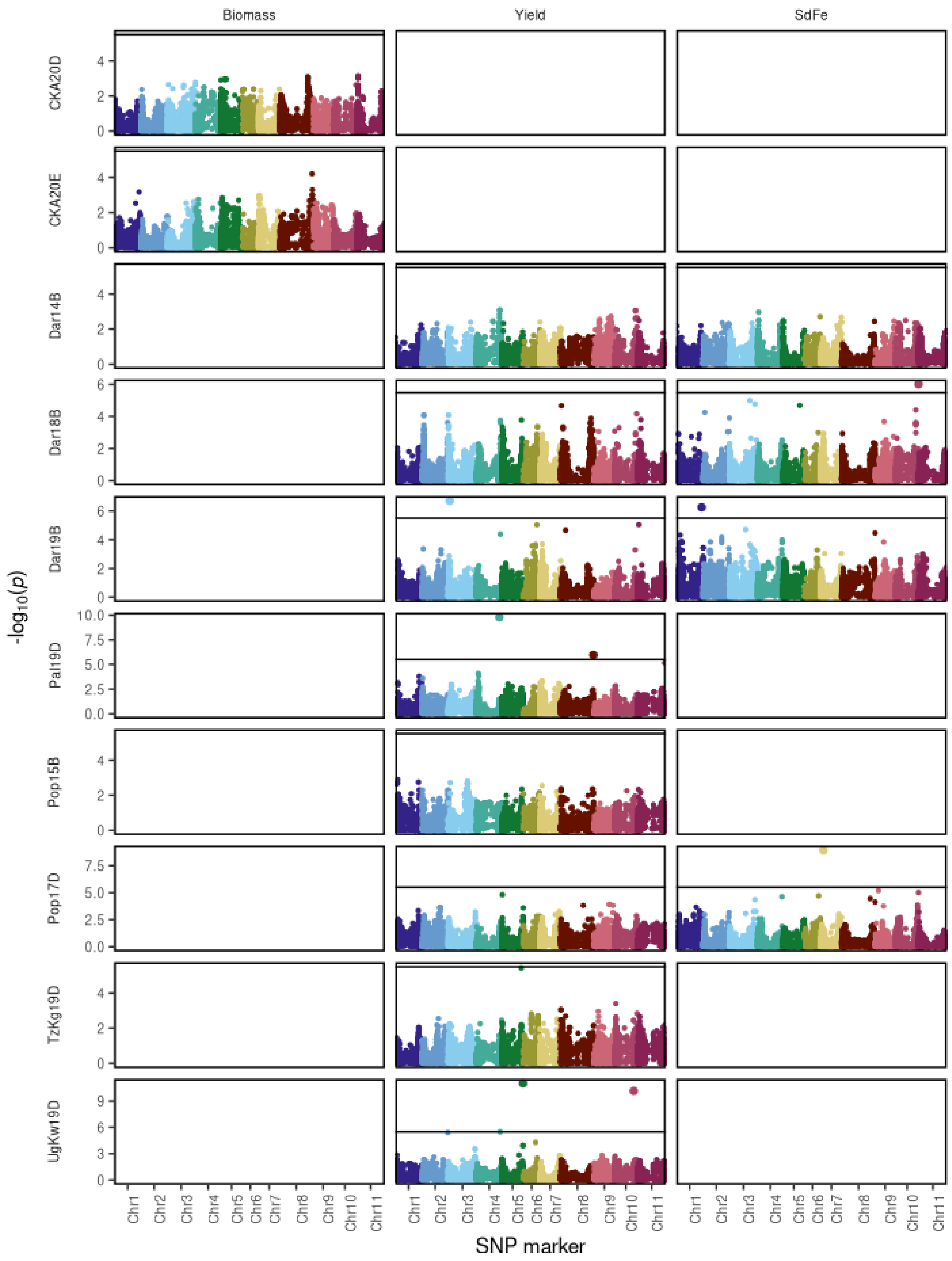

Supplementary Figure 8: The genome-wide association study (GWAS) for biomass, grain yield and seed iron concentration (SdFe) of all available climbing bean population (VEC) trials. Manhattan plots showing the $-log_{10}(p)$ for every marker-trait association. The SNP markers are displayed according to their physical position on the genome. The Bonferroni-corrected significance level is indicated at 5% with a solid line. The VEC population was phenotyped in ten trials, of which six field trials were carried out with the complete population (n=290; Keller et al., 2022a), two field trials included 43 additional climbing bean lines (Dar14A and Pop15B, n=93; Barbosa et al., 2018) and two trials in the greenhouse (CKA20D, n=86 and CKA20E, n=80; this study). Trials were abbreviated based on the location Darién (Dar), Palmira (Pal), Popayán (Pop) in Colombia, Kagera in Tanzania (TzKg), or Kawanda in Uganda (UgKw), the year and the season (A to D).

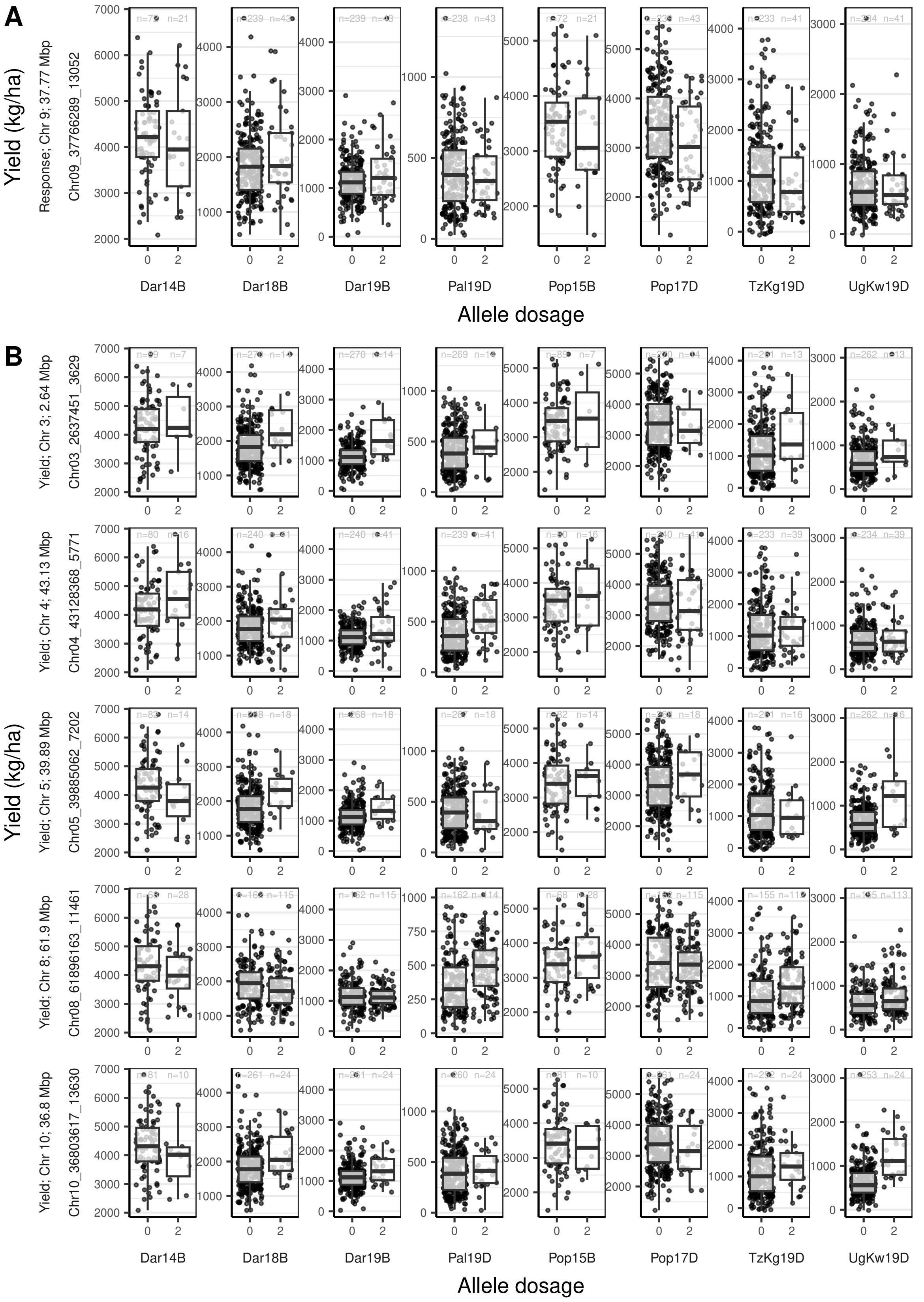

Supplementary Figure 9: Allele dosage effect for A: response of F_q'/F_m' to PPFR ($Response_{G:PPFR}$) and B: yield of significantly associated molecular marker identified in trials with the climbing bean population (VEC). The VEC population was phenotyped in ten trials, of which six field trials were carried out with the complete population (n=290; Keller et al., 2022a) and two field trials included 43 additional climbing bean lines (Dar14A and Pop15B, n=93; Barbosa et al., 2018). Trials were abbreviated based on the location Darién (Dar), Palmira (Pal), Popayán (Pop) in Colombia, Kagera in Tanzania (TzKg), or Kawanda in Uganda (UgKw), the year and the season (A to D).
